# Comment on: Association between anemia and depression: results from NHANES 2005–2018 and Mendelian randomization analyses

**DOI:** 10.1007/s00277-023-05496-9

**Published:** 2023-10-21

**Authors:** Tianyu Si, Xiaolin Ma, Wenwei Zhu, Yongming Zhou

**Affiliations:** Present Address: Department of Hematology, Yueyang Hospital of Integrated Traditional Chinese and Western Medicine, Shanghai University of Traditional Chinese Medicine, 110 Ganhe Road, Shanghai, China

Dear Editor

The current research on the correlation between anemia and depression has yielded inconsistent results, leading to discussions and analyses among researchers. We are interested in reading the study conducted by Wang et al., which was published in advance on July 22, 2023, in the *Annals of Hematology* [1]. This cross-sectional study aimed to evaluate the relationship between anemia/hemoglobin levels and depression among 29,391 participants from the National Health and Nutrition Examination Survey (NHANES) conducted between 2005 and 2018. According to the findings of the study, no causal relationship between anemia and depression was found. We conducted a similar study, analyzing 32,658 participants from the NHANES from 2005 to 2018. The specific selection process is detailed in Fig. 1. Using multiple linear regression analysis, we evaluated anemia and potential confounding factors, including gender, age, race, smoking status, education, and BMI, and obtained similar results. However, after conducting a stepwise regression analysis, we found that gender had a significant impact on the relationship between hemoglobin and depression. Therefore, further analysis was carried out on gender subgroups, as shown in Table 1. It was observed that a strong positive correlation between hemoglobin and depression was present in males: in the adjusted model, participants with mild anemia had an 82% increased risk of developing depression compared to non-depressed participants (*P* < 0.001), while participants with moderate to severe anemia had a 105% increased risk (*P* = 0.010). However, no statistically significant findings were observed in the female subgroup (*P* > 0.05).

**Fig. 1:**
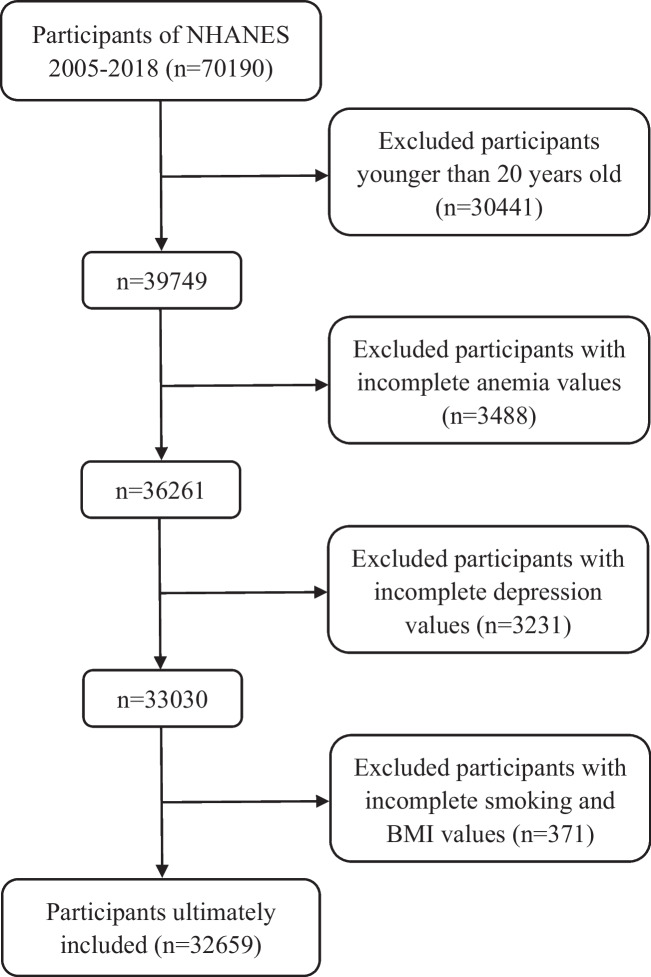
The flowchart of participants. Abbreviation: *BMI*, body mass index

**Table 1:** Association between hemoglobin and depression in multiple regression model

	Non-adjusted model		Model I		Model II		Model III	
	OR (95% CI)	*P*-value	OR (95% CI)	*P*-value	OR (95% CI)	*P*-value	OR (95% CI)	*P*-value
Hemoglobin	0.90 (0.87, 0.93)	< 0.001	1.01 (0.97, 1.06)	0.6	0.95 (0.91, 0.99)	0.027	0.95 (0.90, 0.99)	0.024
Normal	ref		Ref		Ref		Ref	
Mild anemia	1.26 (1.06, 1.50)	0.010	1.13 (0.94, 1.35)	0.2	1.23 (1.03, 1.47)	0.024	1.22 (1.02, 1.46)	0.033
Moderate to severe anemia	1.57 (1.27, 1.93)	< 0.001	1.22 (0.98, 1.53)	0.079	1.22 (0.97, 1.53)	0.092	1.22 (0.96, 1.54)	0.10
Stratified by gender								
Hemoglobin (male)	0.90 (0.84, 0.97)	0.004	0.91 (0.84, 0.98)	0.019	0.88 (0.81, 0.95)	< 0.001	0.88 (0.81, 0.95)	0.001
Normal	ref		Ref		Ref		Ref	
Mild anemia	1.86 (1.40, 2.48)	< 0.001	1.83 (1.36, 2.46)	< 0.001	1.80 (1.33, 2.44)	< 0.001	1.82 (1.35, 2.47)	< 0.001
Moderate to severe anemia	2.24 (1.31, 3.83)	0.003	2.14 (1.24, 3.69)	0.007	2.02 (1.18, 3.46)	0.011	2.05 (1.20, 3.53)	0.010
Hemoglobin (female)	1.06 (1.00, 1.13)	0.069	1.10 (1.03, 1.18)	0.007	1.01 (0.95, 1.08)	0.7	1.01 (0.95, 1.08)	0.8
Normal	Ref		Ref		Ref		Ref	
Mild anemia	0.94 (0.74, 1.18)	0.6	0.87 (0.69, 1.11)	0.3	0.99 (0.78, 1.25)	> 0.9	0.96 (0.76, 1.21)	0.7
Moderate to severe anemia	1.21 (0.95, 1.53)	0.11	1.08 (0.84, 1.39)	0.5	1.07 (0.83, 1.39)	0.6	1.08 (0.83, 1.40)	0.6

Model I: adjusted for sex, age, and race (age and race only by gender)

Model II: adjusted for the variables in model I plus smoking status and education

Model III: adjusted for the variables in model II plus BMI

Our study demonstrates a causal relationship between hemoglobin and depression, but this relationship is only statistically significant in males. This is supported by a previous study conducted in Japan [2]. There are multiple factors that influence depression in females, such as pregnancy, menstrual issues, and other physiological factors [3, 4]. Research has also indicated a negative correlation between hemoglobin and depression among female students at Tehran University of Medical Sciences in Iran [5], suggesting that controlling for certain confounding factors can amplify this association. Although the previous study [1] concluded a lack of significant causal relationship, existing evidence still leans towards a direct correlation between hemoglobin and depression [3, 6, 7]. Therefore, it is crucial to pay attention to the psychological status of anemic patients in clinical practice.

## References

[1] Wang Y, Guo D, Sui C, Qu Z, He G, Meng H, Duan Y, Zhang X, Lan L, Wang C, Liu X (2023). Association between anemia and depression: results from NHANES 2005–2018 and Mendelian randomization analyses. Ann Hematol.

[2] Yi S, Nanri A, Poudel-Tandukar K, Nonaka D, Matsushita Y, Hori A, Mizoue T (2011). Association between serum ferritin concentrations and depressive symptoms in Japanese municipal employees. Psychiatry Res.

[3] Kang SY, Kim HB, Sunwoo S (2020). Association between anemia and maternal depression: a systematic review and meta-analysis. J Psychiatr Res.

[4] McGrath M, Quint EH, Weyand AC (2021). Depression in adolescents and young adults with heavy menstrual bleeding in a referral clinic setting. Am J Hematol.

[5] Vahdat Shariatpanaahi M, Vahdat Shariatpanaahi Z, Moshtaaghi M, Shahbaazi SH, Abadi A (2007). The relationship between depression and serum ferritin level. Eur J Clin Nutr.

[6] Hidese S, Saito K, Asano S, Kunugi H (2018). Association between iron-deficiency anemia and depression: a web-based Japanese investigation. Psychiatry Clin Neurosci.

[7] Vulser H, Wiernik E, Hoertel N, Thomas F, Pannier B, Czernichow S, Hanon O, Simon T, Simon JM, Danchin N, Limosin F, Lemogne C (2016). Association between depression and anemia in otherwise healthy adults. Acta Psychiatr Scand.

